# Supplementary material for: Meeting the Physical Activity Recommendations and Its Relationship with Obesity-Related Parameters, Physical Fitness, Screen Time, and Mediterranean Diet in Schoolchildren
Source: Children (Basel). 2020 Nov 28;7(12):263. doi: 10.3390/children7120263 (PMC7761332; doi:10.3390/children7120263)
Supplement: Supplementary file 1 [file children-07-00263-s001.pdf]

Table S1. Proportion of participants meeting the physical activity recommendations according to the different dependent variables of study.

Variables	Boys	Girls
**ST**		
High ST	35 (43.8)	4 (30.8)
Low ST	45 (56.3)	9 (69.2)
**Adherence to the MD**		
Low MD	19 (23.8)	5 (38.5)
Moderate MD	52 (65.0)	7 (53.8)
High MD	9 (11.3)	1 (7.7)
**Global PF**		
Low Global PF	27 (33.8)	2 (15.4)
Medium Global PF	24 (30.0)	8 (61.5)
High Global PF	29 (36.3)	2 (23.1)
**BF**		
High adiposity	38 (47.5)	6 (46.2)
Low adiposity	42 (52.5)	7 (56.3)
**WHtR**		
Abdominal obesity	10 (12.5)	2 (15.4)
No abdominal obesity	70 (87.5)	11 (84.6)
**BMI**		
Obesity	20 (25.0)	2 (15.4)
Overweight	25 (31.3)	5 (38.5)
Normal weight	35 (43.8)	6 (46.2)

Data expressed as number (percentage). BF: Body fat; BMI: Body mass index; MD: Mediterranean Diet; PF: Physical fitness; ST: Screen time; WHtR: Waist-to-height ratio. Adjusted by age, type of schooling and area of residence.
